# Effectiveness of 38% Silver Diamine Fluoride in Reducing Dentine Hypersensitivity on Exposed Root Surface in Older Chinese Adults: Study Protocol for a Randomised Double-Blind Study

**DOI:** 10.3390/dj10100194

**Published:** 2022-10-19

**Authors:** Alice Kit Ying Chan, Manisha Tamrakar, Chloe Meng Jiang, Yiu Cheung Tsang, Katherine Chiu Man Leung, Chun Hung Chu

**Affiliations:** Faculty of Dentistry, The University of Hong Kong, Hong Kong, China

**Keywords:** older adults, elderly, silver diamine fluoride, dentin, hypersensitivity, prevention, oral health

## Abstract

Background: Dentine hypersensitivity on an exposed root surface induces pain, affects daily oral hygiene practice, limits dietary choices and negatively affects quality of life. Silver diamine fluoride is marketed in the United States as a desensitising agent, but well-designed clinical trials are limited. This study evaluates the anti-hypersensitivity effect of silver diamine fluoride on hypersensitive teeth due to an exposed root surface in older Chinese adults. Methods/design: We will conduct a randomised double-blind clinical trial with a sample size of at least 148 Chinese older adults aged 65 or above who have dentine hypersensitivity due to an exposed root surface. We will collect written consent before the study. A trained examiner will examine the participants’ teeth with a blast of compressed air from a 3-in-1 syringe. Those adults who report a self-perceived sensitivity score (SS) (0 to 10) of 8 or more on at least one tooth with an exposed root surface will be recruited. The recruited older adults will be randomly allocated into two groups using a block randomisation of six. Group 1 participants will receive the application of 38% silver diamine fluoride solution every 4 weeks. Group 2 participants will receive the application of 5% potassium nitrate solution every 4 weeks. Dietary advice, oral hygiene instruction and fluoride toothpaste at 1450 ppm will be provided to participants in both groups. The same trained examiner will perform follow-up examinations for the participants and determine the dentine hypersensitivity in SS of the most hypersensitive tooth (with the highest pre-treatment SS) immediately after the intervention and at 4-week and 8-week intervals. Discussion: There is no consensus on the standard of care for a professionally applied desensitising agent in older adults. This trial will provide evidence for clinicians to devise an effective dental care plan for older adults with dentine hypersensitivity. Trial registration: NCT05392868 Registered on 22 May 2022.

## 1. Background

Caries, periodontal disease and tooth wear are the most common dental problems in older adults [1]. They are the typical causes of dentine hypersensitivity. In 2012, the prevalence of dentine hypersensitivity was 35% among the general population in China, with 38% in older adults aged 60–69 [2]. In 2011, almost half (48%) of the non-institutionalised older adults aged 65–74 in Hong Kong suffered from dentine hypersensitivity [3]. Dentine hypersensitivity is characterised by sharp, short-lasting pain originating from the pulpal tissues in healthy pulp resulting from a stimulus, which can be thermal (hot or cold), electrical, mechanical, osmotic (sweet or sour) or chemical [4]. The mechanism of dentine hypersensitivity is unclear; however, the most common proposed theory is the hydrodynamic theory. The theory suggests that changes in the fluid flow in dentinal tubules due to changes in temperature and air pressure cause hypersensitivity. The fluid movement triggers pain receptors at the nerve endings located at the pulpal aspect to elicit nerve impulses, thereby inducing pain [5]. People who perform an incorrect tooth-brushing technique, have periodontal disease or have recently received periodontal therapy are at risk of developing dentine hypersensitivity [6,7]. Dentine hypersensitivity induces pain and affects the daily oral hygiene practice, which further jeopardises periodontal health [8]. It also limits dietary choices and negatively affects quality of life [8,9].

Managing dentine hypersensitivity begins with obtaining the correct diagnosis through history taking and clinical examination [10]. The aetiological factors should then be addressed by providing proper oral hygiene instruction and dietary advice [10]. The use of desensitising agents can relieve pain. Two modes of treatment action of desensitising agents exist. The first one is by occluding the dentinal tubules; the second is by modifying or blocking the pulpal nerve response [11]. Desensitising agents can be self-applied daily or professionally applied at regular intervals. Desensitising toothpaste incorporated with potassium nitrate is a convenient self-applied method. This treatment effectively manages dentine hypersensitivity; however, it often takes 4–8 weeks to achieve pain relief [11,12]. Clinicians can apply desensitising agents on exposed dentine regularly. Several agents are available, such as sodium fluoride varnish and silver diamine fluoride (SDF) solution [12]. However, there is limited evidence on the clinical efficacy of using professionally applied desensitising agents in reducing dentine hypersensitivity [11].

In 1974, potassium nitrate was introduced as a desensitising agent for in-office therapy to manage dentine hypersensitivity [13]. Currently, it is one of most frequently used desensitising agents, since the American Dental Association accepted it in 1986 [14]. Its mechanism of treatment action is believed to involve depolarising the cellular membrane of the nerve terminal and, hence, decreasing the nerve response [12]. The efficacy of potassium nitrate in both self-applied toothpastes and a professionally applied solution has been observed in clinical trials [13,15]; hence, it has been used as a conventional method in managing dentine hypersensitivity. However, studies have reported conflicting results, and the efficacy of potassium nitrate in reducing dentine sensitivity has recently been questioned [11].

SDF is used as a professionally applied desensitising and caries-arresting agent [16]. The United States Food and Drug Administration cleared SDF in 2014 to treat dentine hypersensitivity [17]. A study demonstrated that SDF occluded the dentinal tubules on the exposed dentine surface [18]. This explained its desensitising effect. However, there are limited well-designed clinical trials that investigate the effectiveness of SDF in reducing dentine hypersensitivity in older adults [19,20].

## 2. Objective

This study’s objective is to evaluate the desensitising effect of the topical application of 38% SDF solution on the exposed root surfaces of hypersensitive teeth in older Chinese adults.

## 3. Hypothesis

The hypothesis under investigation is that the topical application of 38% SDF solution is more effective than 5% potassium nitrate solution in desensitising hypersensitive teeth with an exposed root surface in older Chinese adults.

## 4. Methods/Design

### 4.1. Trial Design

This is a randomised, single-centre, double-blind clinical trial with two parallel arms. The trial’s design and reporting follows the Standard Protocol Items: Recommendations for Interventional Trials (SPIRIT) statement (Supplementary Material S1) [21].

### 4.2. Participant Timeline

**Figure 1:**
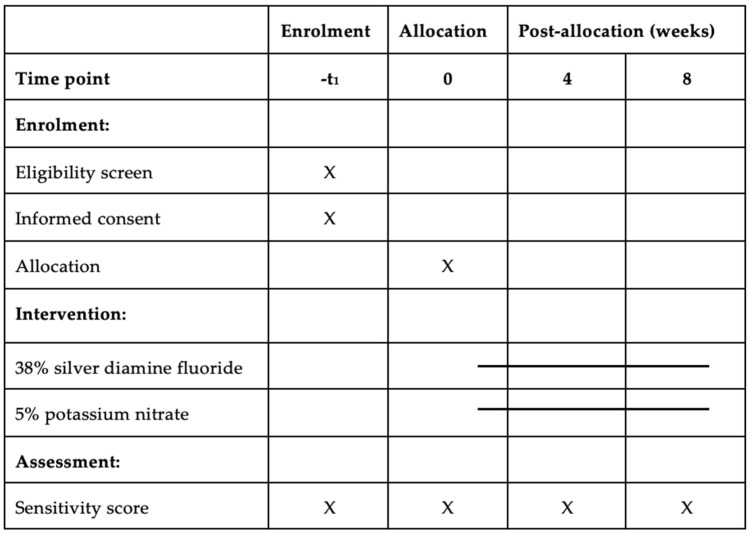
The schedule of enrolment, intervention and assessments.

A SPIRIT figure is used to describe the trial schedule of enrolment, intervention and assessment and is presented in Figure 1.

### 4.3. Study Setting

This clinical trial will be conducted in the Clinical Research Centre in the Faculty of Dentistry of the University of Hong Kong.

### 4.4. Participants

**Figure 2:**
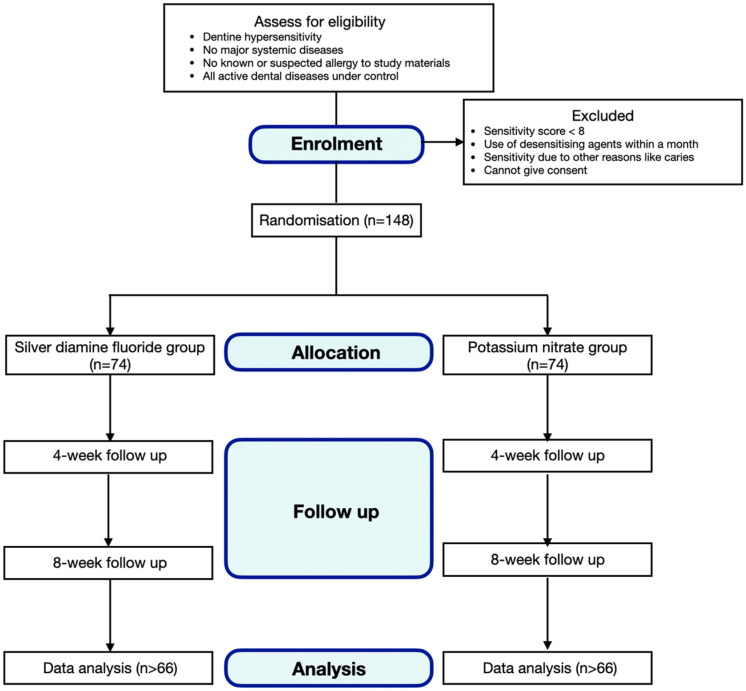
Consort flow diagram.

We will recruit healthy older adults, aged 65 or older, with dentine hypersensitivity. They should have no known or suspected allergies to the study ingredients or materials, and they should have all active dental diseases under control. Older adults will be excluded if they have been using any desensitising agent within the past month, have major systemic diseases such as cancer, have dentine hypersensitivity due to other dental conditions such as caries or cannot give written consent. A research assistant will call older adults via phone. Those who have experienced symptoms of dentine hypersensitivity (such as sharp pain when eating cold or sour food) within 4 weeks will be invited for a dental examination. Figure 2 shows the consort flow diagram.

### 4.5. Informed Consent

A research assistant will explain the study’s purpose and procedures to all potential older adults who meet the inclusion criteria and the assistant will obtain their written consent (Supplementary Material S2).

### 4.6. Baseline and Follow-Up Clinical Examinations

A trained dentist will perform baseline and follow-up clinical examinations and assessments of dentine hypersensitivity. We will collect demographic background information, such as age, sex, dietary habit and denture use. A research assistant will measure the compressed cold air pressure and temperature of all the 3-in-1 syringes to be used in the Clinical Research Centre via a digital pressure indicator (DPI 2600847b) and a thermocouple digital sensor/thermometer (KM2013c) before all baseline and follow-up clinical examinations. The acceptable ranges of compressed cold pressure and temperature are 65 to 75 psi and 18 to 24 °C, respectively [8].

**Figure 3:**
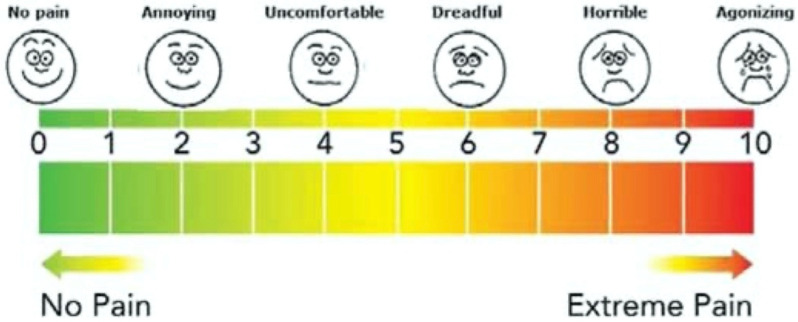
Sensitivity score scale table.

A trained examiner will measure the oral hygiene status of the older adults via the visible plaque index (VPI) and will record it as 0 for the absence of plaque and 1 for the presence of plaque [22]. The trained examiner will dry and clean all tooth surfaces with a piece of gauze. Cotton rolls will isolate hypersensitive teeth due to exposed root surface. They will then be assessed for dentine hypersensitivity using compressed air delivered from a 3-in-1 syringe placed approximately 1 cm away from and perpendicular to the exposed root surface for 5 s [23]. The older adult will give a self-perceived sensitivity score (SS) from 0 to 10 to all teeth under assessment for dentine hypersensitivity [8]. An SS of 0 will indicate no discomfort, whereas an SS of 10 will indicate maximum pain causing great distress to the subject (Figure 3) [24].

We will exclude older adults with a pre-treatment SS of less than 8 from this study. For each older adult, we will select the most hypersensitive tooth (the highest SS) due to exposed root surface for the assessment of dentine hypersensitivity in follow-up visits. A dental periodontal graduated probe at the nearest mm will measure the maximum amount of gingival recession of the selected hypersensitive tooth. The same trained examiner will perform the clinical examination and assessment of dentine hypersensitivity immediately after treatment and at 4-week and 8-week follow-up visits, using the same tools and procedures as in the baseline examination. We will re-examine 10% of the older adults later on the same day to assess the intra-examiner agreement at the baseline and follow-up examinations. To promote the follow-up rate, a research assistant will remind all older adults of their follow-up appointment schedule. If they are absent at the follow-up visit, another timeslot will be provided within the same week.

### 4.7. Intervention

After the baseline or follow-up clinical examinations, the eligible older adults will receive either 38% SDF solution (Saforide 38%, Morita, Osaka, Japan) or 5% potassium nitrate solution (Potassium Nitrate, Sigma-Aldrich, Darmstadt, Germany) on the exposed root surface of the selected hypersensitive tooth. If the adjacent tooth or teeth are also hypersensitive, the adjacent tooth or teeth will also receive therapy. An independent operator will dry the selected hypersensitive tooth and use a micro-brush to apply either 38% SDF solution or 5% potassium nitrate solution for 60 s [25], according to the assigned treatment group, on the exposed root surface. The SDF and potassium nitrate will be applied at baseline and at the 4-week follow-up visit after clinical examination. All eligible older adults will receive dietary advice, standardised oral hygiene instruction, a toothbrush and a regular fluoridated toothpaste (1450 ppm) after baseline examination until the end of the study period. We will instruct all older adults not to eat or drink for 30 min after SDF or potassium nitrate application. Because a dentist will professionally apply the intervention, there will be no concern regarding adherence to the intervention for both groups. Applying SDF or potassium nitrate solution will not affect the participants’ usual care pathways, such as medication use. They are prohibited from receiving other dental treatments for dentine hypersensitivity during the study period. In addition, they must follow the oral hygiene instruction received at the baseline visit.

### 4.8. Randomisation, Treatment Group Allocation and Allocation Concealment

A research assistant will randomly allocate eligible older adults who have at least 1 hypersensitive tooth with an SS of 8 or more due to exposed root surface into 2 intervention groups with a block randomisation of 6 and with a 1:1 ratio. A statistician will generate a random number sequence in a computer. The 2 intervention groups are as follows:

- SDF (test) participants will receive topical application of 38% SDF solution on the exposed root surface of the most hypersensitive tooth every 4 weeks;
- Potassium nitrate (positive control) participants will receive topical application of 5% potassium nitrate solution on the exposed root surface of the most hypersensitive tooth every 4 weeks.

There will be no negative control group for ethical reasons. A research assistant will keep the random number sequence in opaque sealed envelopes and conceal the allocation sequence until interventions are assigned.

### 4.9. Blinding

The examiner and the participating older adults will be blinded to the group allocation throughout the study. An independent operator will apply SDF or potassium nitrate solution after clinical examination. If the older adult requests the treatment history, unblinding is allowed. An unblinded research assistant will disclose treatment allocation to that older adult, who will then be excluded from the study.

### 4.10. Outcome Measure

The primary outcome will be the change in dentine hypersensitivity of the most hypersensitive tooth in response to compressed air blast stimuli. This will be measured as the change in SS from the baseline visit to the 8-week follow-up visit after the intervention, to evaluate the anti-hypersensitivity effect of SDF and potassium nitrate solution.

### 4.11. Sample Size Calculation

Based on a previous clinical trial [8], we estimate that the percentage of change in SS in the test and positive control groups will be 52% and 40%, respectively, with a common standard deviation of 22% and 23%, respectively [8]. Using a power of 0.8 and a statistical significance of 0.05, the minimum number of older adults needed for the study with a dropout rate of 10% was calculated to be 148, with 74 older adults per group, which was determined via the Mann–Whitney test using the software G*Power 3.1 (Franz Faul, Kiel University, Kiel, Germany) [26].

### 4.12. Data Management

Two research assistants will enter the data collected into an Excel file (by double entry) independently, and we will then compare the data to correct errors. A statistician will inspect the data entry, data checking and analysis for this study.

### 4.13. Data Analysis

We will apply the statistical software SAS for Windows (SAS Institute Inc., Cary, NC, USA) and SPSS for Windows (IBM Corporation, Armonk, NY, USA) for data analyses. The data will be assessed for a normal distribution using the Shapiro–Wilk test for normality. A chi-squared test will be used to determine the difference in distribution of gender and tooth type between the 2 groups. A Mann–Whitney U test will be used to study the pre- and post-treatment SS and the reduction in SS of the treatment group if these data are not normally distributed, whereas a t-test will be used instead if these data are normally distributed. We will report the medians and interquartile ranges for all of the continuous variables to provide information on the data distribution. All statistical tests will be 2-tailed, and the significance level will be set at 0.05. This study will use the intention-to-treat principle for analysis. However, if the number of older adults who withdraw from the study is significantly different between test and positive control groups at the 8-week follow-up visit, we will use a per-protocol approach for analysis and only older adults who have completed the 8-week follow-up will be included in the results. We will also study independent variables, such as demographic background and VPI, which may modify the treatment effect.

### 4.14. Ethical Consideration

We sought ethical approval from the local Institutional Review Board (UW22-517). Written consent will be obtained from all participating older adults after explaining the study’s purpose and procedures. All participating older adults will be allowed to withdraw from the study at any time through informing the primary investigator. Withdrawal from the study will not affect their right to receive other dental care, such as oral health education. The study will present a minimal risk to the participating older adults. All the field workers will receive professional training to minimise the risk. We will inform participants via the consent form that if they are harmed by taking part in this clinical trial, no compensation will be provided. If one life-threatening case is found or more than 30% of the participants suffer from severe systemic side effects, this clinical trial will cease immediately. All the personal information about potential and enrolled participants will be confidential in a personal computer with a password, and only the investigators will have access to it.

## 5. Discussion

This is a phase II, randomised, two-parallel-arm, double-blind clinical trial to study the effect of 38% SDF solution in reducing dentine hypersensitivity on hypersensitive teeth with an exposed root surface in older Chinese adults aged 65 or older. Considering that SDF forms deposits in dentinal tubules, causing tubular occlusion [18], our hypothesis is that 38% SDF is more effective than 5% potassium nitrate solution in reducing dentine hypersensitivity on hypersensitive teeth with an exposed root surface in older Chinese adults aged 65 or older.

Regarding the control used in this study, we will choose a positive control instead of a negative control due to ethical issues. Currently, no desensitising agent has yet been proven effective in managing dentine hypersensitivity over other desensitising agents; therefore, no product or agent was recognised as a gold standard for the positive control in clinical trials investigating an anti-hypersensitivity effect [27]. We will use potassium nitrate in this study due to its long history as a desensitising agent, the evidence showing its clinical efficacy in reducing dentine hypersensitivity and its widespread use in clinical dentistry. In this study, potassium nitrate will be prepared as a 5% solution to blind the participants and eliminate the placebo effect. A placebo effect may be anticipated in a clinical trial for assessing pain outcomes, as stated in a systematic review [28]. The desensitising agents used in this study will be in the form of a solution, which is difficult to contain within the applied tooth surface. Therefore, we will use a parallel rather than split-mouth design to avoid the carry-over effect.

A compressed cold air test, tactile test or thermal stimulus is commonly used to assess dentine hypersensitivity in clinical trials [11]. Although the guidelines from Holland et al. in 1997 recommended that dentine hypersensitivity should be assessed by two independent stimuli, separated by a sufficient time interval, to minimise the interaction between the stimuli, no guidelines provide the suggested time interval [27]. Therefore, we will apply only one test stimulus in this study to assess dentine hypersensitivity, so as to avoid any interaction between stimuli and to simplify the procedure for the older adults. Compressed cold air will be used because 3-in-1 syringes are commonly available in clinical practice. Both the pressure and temperature of 3-in-1 syringes can be standardised and monitored during the study to minimise variations. In addition, the procedure is simple, non-invasive and older adults can easily tolerate it.

Teeth or carious lesions under investigation, found in the same individual, will impose a clustering effect due to the within-group similarity [29]. The clustering effect reduces the precision and statistical power and widens the confidence intervals [29]. Therefore, only the most hypersensitive tooth (the tooth with the highest pre-treatment SS) will be selected and recorded for data analysis to avoid the clustering effect.

Stimulus-based or response-based assessments can evaluate dentine hypersensitivity [27]. Stimulus-based assessment is performed via applying a stimulus in an intensity to evoke pain, and it is used to measure the pain threshold; response-based assessment is performed by subjectively evaluating the pain stimuli produced, and it is used to estimate pain severity [27]. This study aims to evaluate whether SDF can reduce dentine hypersensitivity; hence, we will utilise a response-based assessment to investigate the change in pain severity that the participants perceive. In the response-based method, the visual analogue scale, numeric rating scale, verbal descriptor scale and faces pain scale are generally used for measurement [30]. Of these, the verbal descriptor scale was recommended as the scale of choice for evaluating pain intensity among older adults [30]. The SS that we will use in this study is modified according to the verbal descriptor scale, with the addition of adjective words representing different levels of pain intensity, so as to enhance the older adults’ understanding [8]. The outcome will be the change in SS from the baseline visit to the 8-week follow-up visit.

Diet and oral hygiene practice will influence dentine hypersensitivity [12]. We will provide all participants with dietary advice and standardised oral hygiene instructions before the intervention, to avoid any modification to the outcome measure. The older adults will receive a clinical examination and compressed cold air test only. Both SDF and potassium nitrate solution have been approved as safe for clinical use, and the related procedure for clinical application is simple and non-invasive. Therefore, this study will have limited ethical concerns. 

## 6. Trial Status

This clinical trial was registered on ClinicalTrials.gov (United States) under the registration number NCT05392868 on 22 May 2022. This protocol is version 1, written on 21 July 2022. Participant recruitment began on 1 September 2022 and this study is expected to end on 30 April 2023.

## Data Availability

Not applicable.

## Supplementary Materials

The following information can be downloaded at: https://www.mdpi.com/article/10.3390/dj10100194/s1, S1: SPIRIT 2013 checklist; S2: Information sheet and consent form.

## Roles and Responsibilities

A professor (C.H.C.) in the Faculty of Dentistry of the University of Hong Kong leads and supervises the trial steering committee. The committee will hold a meeting once per month through a face-to-face or online conference to monitor the trial’s progress. The Faculty of Dentistry of the University of Hong Kong supports this trial. The Faculty of Dentistry has reviewed the protocol and will monitor the trial’s progress. The Faculty of Dentistry of the University of Hong Kong is the sponsor but has no influence on the conducting of this trial. A statistician from the Central Research Centre in the faculty will monitor the data. A progress report will be provided to all relevant parties, including the Institutional Review Board and the Central Research Centre. The committee will inform all the relevant parties about any amendments made to the protocol from time to time.
