# Supplementary material for: Effectiveness of 38% Silver Diamine Fluoride in Reducing Dentine Hypersensitivity on Exposed Root Surface in Older Chinese Adults: Study Protocol for a Randomised Double-Blind Study
Source: Dent J (Basel). 2022 Oct 19;10(10):194. doi: 10.3390/dj10100194 (PMC9600009; doi:10.3390/dj10100194)
Supplement: Supplementary file 1 [file dentistry-10-00194-s001.zip › dentistry-1911857-supplementary/S2 Information sheet and consent form.pdf]

INFORMATION SHEET

Research Title

Effectiveness of 38% silver diamine fluoride in reducing dentine hypersensitivity on exposed root surface in older Chinese adults: a randomized double-blind study

Invitation

You are being invited to take part in a research study. Before you decide to take part or not it is important for you to understand why the research is being done and what it will involve. Please take time to read the following information carefully and discuss it with friends, relatives and your family doctor/dentist if you wish. Ask us if there is anything which is not clear or if you would like to have more information. Take time to decide whether or not you wish to take part.

Background of study

Dentine hypersensitivity is a common oral complaint among older adults. It impacts daily oral care, affects dietary choices, and lower the quality of life. It can be managed by using desensitizing agents. Silver diamine fluoride (SDF) and potassium nitrate (KNO_3) are two available desensitizing agents that can be applied professionally.

Aims

The aim of the study is to investigate the anti-hypersensitivity effect of silver diamine fluoride (SDF) and potassium nitrate (KNO_3) in older Chinese adults.

Why am I chosen?

You are at 65 years old or above, generally healthy, with no known or suspected allergy to study ingredients, and have active dental diseases under control but reported dentine hypersensitivity on exposed root surface. Therefore, you are invited to take part in this research project which will be conducted in Prince Philip Dental Hospital.

Do I have to take part?

It is up to you to decide whether or not to take part. If you do decide to take part you will be given this information sheet to keep and be asked to sign a consent form. If you decide to take part you are still free to withdraw at any time without giving a reason. This will not affect the standard of care you receive in the Prince Philip Dental Hospital (PPDH). If you receive any other dental treatment for dentine hypersensitivity, you will be excluded in this study without affecting the standard of care you receive in PPDH.

What will happen to me if I take part?

This is an 8-week study. This study will be carried out in the Prince Philip Dental Hospital. At the first visit, you will receive dietary advice and oral hygiene instruction with the provision of toothbrush and toothpaste during the study period. You will receive an oral examination and the hypersensitive teeth will be assessed by a blast of compressed cold air from a 3-in-1 syringe. You will be asked to rate the hypersensitivity on a sensitivity score from '0' as no discomfort to '10' as maximum pain. You will then receive either SDF or KNO_3 treatment on the root surface of the most hypersensitive tooth with a score of 8 or above once every 4 weeks. You will be followed up every 4 weeks. At the follow-up visit, you will receive oral examination and the most hypersensitive tooth will be assessed for sensitivity using the same tools and procedure up to 8-week follow-up. The eligible older adults will be randomly allocated into two intervention groups, SDF or KNO_3 intervention groups, at a ratio of 1:1 with a block randomization of six. You will not know which group you are allocated to, however, if you would like to have the treatment history, unblinding is allowed yet you will then be excluded from the study. It will not affect the standard oral care you receive in PPDH. The first visit will take 20 minutes while the follow-up visits every 4 weeks will take 10 minutes. No radiograph will be taken. No lifestyle or dietary restriction is required during this period. All older adults should not receive any other treatment for dentine hypersensitivity. You should use the toothbrush and toothpaste provided during the study period.

If I take part in this study, how long will this study last?

This study will last for 8 weeks.

What are the benefits of taking part?

The SDF or KNO_3 treatment may relieve dentine hypersensitivity.

What are the risks and side effects of taking part?

You will receive either SDF or KNO_3 treatment on the exposed root surface of the most hypersensitive tooth. If tooth decay is presented, it may be stained black after treatment. The black stain will not fade away, yet it means the tooth decay is successfully stopped from progression. However, if there is a layer of bacteria over the exposed root surface, the bacteria may be killed after the treatment and be stained black. Black stain on bacteria is not permanent and can be removed by professional toothbrushing.

If I take part in this study, do I need to pay extra fees or receive any rewards?

You will not be charged for this study, and you will not receive any rewards.

What if something goes wrong?

If you feel any discomfort and would not like to continue the oral examination or treatment application, all procedures will be stopped immediately. If you are harmed by taking part in this research project, there are no special compensation arrangements. However, if you are harmed due to someone's negligence, then you may have grounds for a legal action. Regardless of this, if you wish to complain about any aspect of the way, you have been approached or treated during the course of this study, the normal health service complaints mechanisms may be available to you.

Will my taking part in this study be kept confidential?

All information which is collected about you during the course of the research will be kept strictly confidential. Any information about you which leaves the hospital will have your name and address removed so that you cannot be recognized from it.

Will my personal information be strictly protected and kept confidential?

We collect personal health information about you. The information that we collect may include your date of birth and gender. We will take steps to protect your personal health information from theft, loss and unauthorized access, copying, modification, use, disclosure and disposal. Steps will also be taken to ensure that everyone who performs services for us protect your privacy and only uses your personal health information for the purposes you have consented to.

Confidentiality

You have the rights of access to personal data and publicly available study results, if and when needed. Under the laws of Hong Kong (in particular the Personal Data (Privacy) Ordinance, Cap 486), you enjoy or may enjoy rights for the protection of the confidentiality of your personal data, such as those regarding the collection, custody, retention, management, control, use (including analysis or comparison), transfer in or out of Hong Kong, non-disclosure, erasure and/or in any way dealing with or disposing of any of your personal data in or for this study. For any query, you should consult the Privacy Commissioner for Personal Data or her office (Tel No. 2827 2827) as to the proper monitoring or supervision of your personal data protection so that your full awareness and understanding of the significance of compliance with the law governing privacy data is assured.

By consenting to participate in this study, you expressly authorize:

- The principal investigator and his research team and the ethics committee responsible for overseeing this study to get access to, to use, and to retain your personal data for the purposes and in the manner described in this informed consent process; and

- The relevant government agencies (e.g. the Hong Kong Department of Health) to get access to your personal data for the purposes of checking and verifying the integrity of study data and assessing compliance with the study protocol and relevant requirements.

What will happen to the results of the research study?
The results would likely be disseminated through local or international journals. Please note that you will not be identified in any report/publication.

Who has reviewed this study?
This study was reviewed and approved by the Institutional Review Board of the University of Hong Kong / Hospital Authority Hong Kong West Cluster.

Contact for further information
Should you need further information, please contact Dr. Chan Kit Ying at 3B16, 3/F, Prince Philip Dental Hospital, 34 Hospital Road, Sai Yin Pun, Hong Kong, Tel: 28580409
On behalf of Faculty of Dentistry, the University of Hong Kong, we would like to thank you for reading this information sheet and for your support for this study.

Dr. Chan Kit Ying, Faculty of Dentistry, the University of Hong Kong
Date:

INFORMED CONSENT FORM

PPDH number: ____________________

Title of study: Effectiveness of 38% silver diamine fluoride in reducing dentine hypersensitivity on exposed root surface in older Chinese adults：a randomized double-blind study

Name of Researcher: Dr Chan Kit Ying

Please tick ✓in the box

1. I confirm that I have read and understood the information sheet dated_____________for the above study and have had the opportunity to ask questions. ☐

2. I understand that my participation is voluntary and that I am free to withdraw at any time, without giving any reason, without my medical care or legal rights being affected. ☐

3. I understand that sections of any of my medical notes may be looked at by responsible individuals from Faculty of Dentistry, The University of Hong Kong or from Institutional Review Board of the University of Hong Kong / Hospital Authority Hong Kong West Cluster. I give permission for these individuals to have access to my records. ☐

4. I agree to take part in the above study. ☐

____________________	____________________	____________________
Name of Participant	Date	Signature
Dr Chan Kit Ying		
Name of Researcher	Date	Signature
